# Acute promyelocytic leukaemia presenting with gingival hypertrophy: an atypical oral presentation

**DOI:** 10.1093/omcr/omag097

**Published:** 2026-06-15

**Authors:** Emmanuel Kissiedu Antiri, Ivy Adwowa Efiefi Ekem

**Affiliations:** Department of Haematology, Cape Coast Teaching Hospital, P.O. Box CT 1363, Cape Coast, Central Region, Ghana; Department of Haematology, University of Cape Coast School of Medical Sciences, Cape Coast, Central Region, Ghana; Department of Haematology, University of Cape Coast School of Medical Sciences, Cape Coast, Central Region, Ghana

**Keywords:** acute promyelocytic leukaemia, gingival hypertrophy, atypical presentation

## Abstract

Acute promyelocytic leukaemia (APL) is a subtype of acute myeloid leukaemia characterised by the PML–RARα fusion gene and a high risk of early mortality if treatment is delayed. Gingival hypertrophy is an uncommon manifestation of APL and is more typically associated with monocytic and myelomonocytic AML. We report a 26-year-old male who presented with a three-week history of progressive gingival enlargement, initially managed as gingivitis for two weeks, resulting in delayed haematological evaluation. Microgranular APL was confirmed by bone marrow morphology and fluorescence in situ hybridisation, demonstrating t(15;17). Although pancytopenia was present, the absence of hyperleukocytosis or overt coagulopathy contributed to an atypical clinical picture. Due to resource constraints, all-trans retinoic acid–based therapy was unavailable, and induction chemotherapy was administered, with a fatal outcome after one week. This case highlights gingival hypertrophy as an atypical early presentation and the impact of delayed diagnosis and limited access to essential therapy.

## Introduction

Acute promyelocytic leukaemia (APL) is a distinct subtype of acute myeloid leukaemia (AML) characterised by the PML-RARα fusion and associated with a high risk of life-threatening coagulopathy. APL occurs in two recognised morphologic forms: the hypergranular and microgranular (hypogranular) variants and constitutes approximately 5–8% of AML cases in younger patients, with a lower frequency in the elderly [1]. Formerly classified as AML-M3 in the French–American–British (FAB) system, APL most commonly arises from the balanced translocation t(15;17)(q24.1;q21.2), resulting in fusion of the promyelocytic leukaemia (PML) gene on chromosome 15 with the retinoic acid receptor-alpha (RARα) gene on chromosome 17 [2]. The resulting PML-RARα oncoprotein promotes leukaemogenesis through transcriptional repression and blockade of myeloid differentiation, leading to accumulation of abnormal promyelocytes [3].

Clinically, patients typically present with manifestations of bone marrow failure, including dizziness, bleeding tendencies, and recurrent infections. In the microgranular variant, hyperleukocytosis may occur and be accompanied by leukostasis symptoms such as dyspnoea, headache, visual disturbance, seizures, or coma. APL, particularly the microgranular variant, is also associated with a high risk of disseminated intravascular coagulation (DIC) and other coagulopathies [4], contributing to early mortality if treatment is delayed. The early initiation of all-trans retinoic acid (ATRA), combined with arsenic trioxide (ATO) and other chemotherapeutic drugs depending on risk category, together with comprehensive supportive care, has transformed APL into a highly curable disease with excellent long-term outcomes [1, 5].

Diagnostic imaging in leukaemia, including APL, serves a supportive role alongside laboratory and bone marrow evaluation. Cross-sectional modalities, including radiography, computed tomography (CT), and magnetic resonance imaging (MRI), are primarily used to detect complications, assess organ involvement, and identify extramedullary disease, and may occasionally suggest an underlying diagnosis in patients with nonspecific presentations. However, imaging does not have a routine role in diagnosis, risk stratification, or surveillance [6]. Functional imaging, particularly 18F-FDG PET/CT, enables whole-body assessment of bone marrow activity and can detect extramedullary involvement, monitor treatment response, and identify relapse or occult disease. Despite these advantages, its role in routine leukaemia management remains evolving and not yet fully established [7].

APL typically involves the bone marrow and peripheral blood, with extramedullary involvement being uncommon [8]. Gingival hypertrophy is more frequently associated with monocytic AML subtypes, particularly acute monocytic leukaemia (AML-M5) and acute myelomonocytic leukaemia (AML-M4) [9]. Gingival hypertrophy in association with APL is rare, with only a limited number of case reports [10, 11]. Retrospective studies from India have reported variable incidence ranging from 5.7% to 20% [10]. Data from Africa remains scarce, and such atypical presentations may be misattributed to primary dental disease, resulting in delayed haematological evaluation. We present a case of microgranular APL presenting with gingival hypertrophy, initially managed as gingivitis, to emphasise the importance of early suspicion, prompt haematological evaluation, and timely initiation of appropriate therapy.

### Case report

A 26-year-old male presented with a three-week history of progressive gum swelling and bleeding, accompanied by haematuria, fever, dizziness, and palpitations. He initially sought care at a peripheral facility and was managed for presumed gingivitis for two weeks without improvement, prompting referral for further evaluation.

On examination, the patient was acutely unwell with marked pallor, extensive gingival hypertrophy with bleeding, and generalised petechial lesions. There was no clinical evidence of lymphadenopathy or organomegaly.

**Figure 1:**
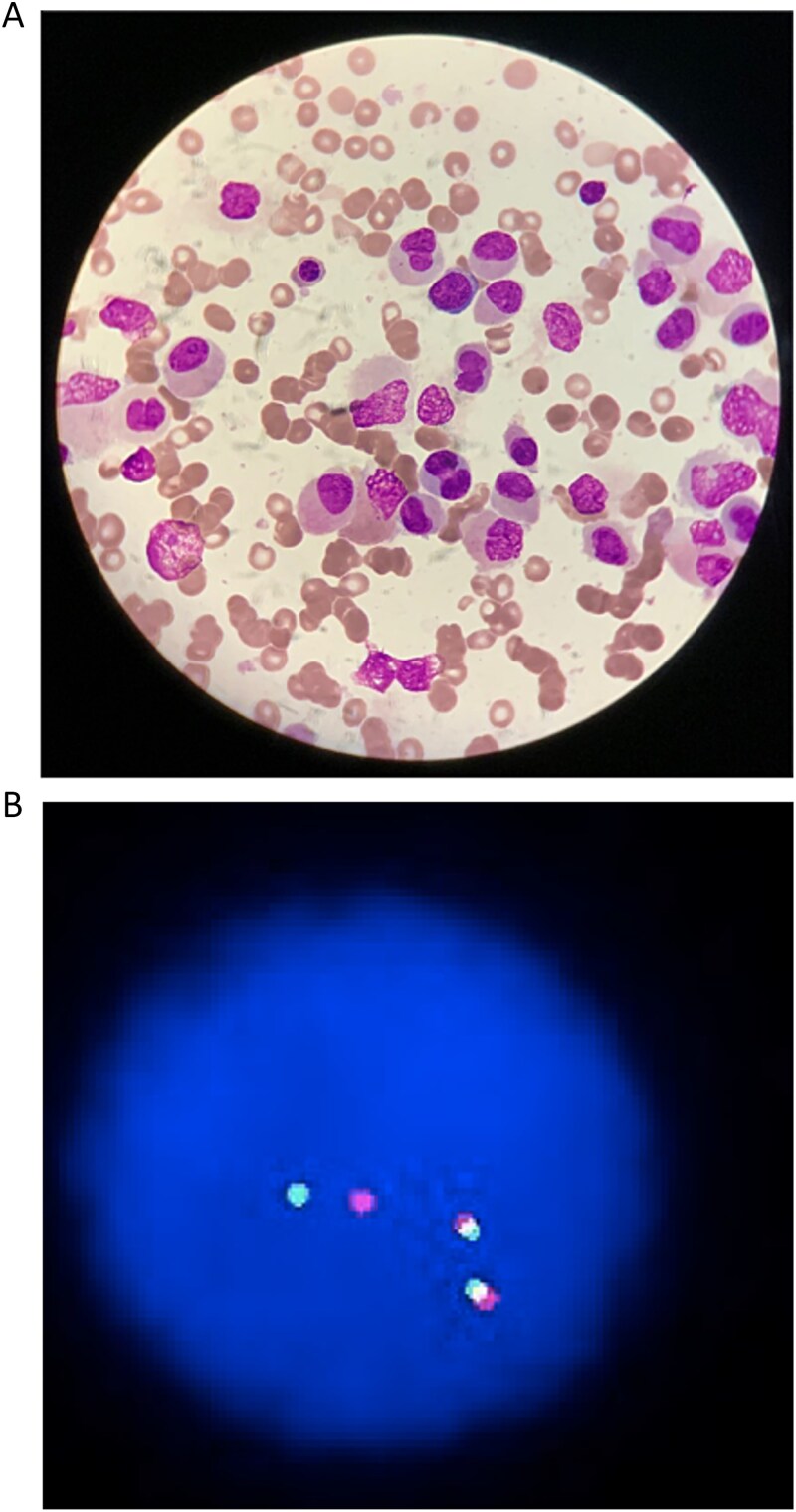
Acute promyelocytic leukaemia (APL) (bone marrow aspirate). (A) Immature myeloid cells, some with bilobed nucleus characteristic of APL, microgranular variant. Fluorescence in situ hybridisation (bone marrow aspirate). (B) The probe hybridises to chromosomes 15q22 (PML gene – Orange) and chromosomes 17q21 (RARα gene – Green), which gives a normal pattern of two orange, two green and a pattern of two fusion (yellow), one orange and one green in t(15;17) positive cases. Orange (PML signal); green (RARα signal); yellow (fusion signal for PML-RARα).

Initial laboratory evaluation revealed pancytopenia on full blood count (FBC) (Table 1). Coagulation studies were within normal limits (Table 2). Additional biochemical investigations demonstrated mild transaminitis and elevated lactate dehydrogenase (Table 3). Urinalysis was unremarkable except for trace proteinuria (+), with otherwise normal parameters. Bone marrow aspiration (BMA) demonstrated features consistent with APL, microgranular variant (Fig. 1A). Flow cytometry showed abnormal myeloid cells expressing CD13, CD33, and cytoplasmic myeloperoxidase (MPO). Fluorescence in situ hybridisation confirmed the presence of the PML-RARα fusion resulting from t(15;17) in 94% of analysed interphase cells (Fig. 1B).

**Table 1:** FBC findings at presentation, during and after therapy.

Parameter	At presentation	Before therapy (Post CRC and platelet transfusion)	During therapy	After therapy	Reference range
Haemoglobin (g/dl)	7.2 ↓	8.5 ↓	6.5 ↓	5.3 ↓	13.0–18.0
WBC (×10^3^/μl)	3.0 ↓	1.41 ↓	1.01 ↓	0.3 ↓	4.0–10.0
Platelets (×10^3^/μl)	7 ↓	31 ↓	16 ↓	10 ↓	150–450
Neutrophils (×10^3^/μl)	0.86↓	0.52↓	0.21↓	0.01 ↓	1.5–8.0

Arrows indicate values below (↓) or above (↑) the reference range. FBC: Full blood count; WBC: White blood cells; Hb: Haemoglobin; CRC: Concentrated red cells. Therapy refers to induction chemotherapy consisting of intravenous cytarabine (170 mg daily for 7 days) and doxorubicin (50 mg daily for 3 days)

**Table 2:** Coagulation profile at presentation.

Parameter	Result	Reference range
PT (sec)	13.0	11–14
INR	1.23	0.9–1.2
APTT (sec)	27.1	27–43

PT: prothrombin time; INR: international normalised ratio; APTT: activated partial thromboplastin time.

**Table 3:** Biochemical and supportive laboratory findings.

Parameter	Value	Reference range
Creatinine (μmol/l)	91.0	53.0–123.8
Urea (mmol/l)	2.65	2.14–7.12
AST (U/l)	46.7 ↑	5.0–34.0
ALT (U/l)	106.8 ↑	10.0–50.0
ALP (U/l)	268.8	53.0–270.0
Total bilirubin (μmol/l)	7.82	3.40–25.70
Direct bilirubin (μmol/l)	2.36	0.00–10.30
Albumin (g/l)	47.5	34.00–50.00
Uric acid (μmol/l)	169.2 ↓	208.3–428.4
LDH (U/l)	332↑	125.0–280

Arrows indicate values below (↓) or above (↑) the reference range. AST: aspartate aminotransferase; ALT: alanine aminotransferase; ALP: alkaline phosphatase; LDH: lactate dehydrogenase.

Due to limited access to ATRA-based therapy, the patient was commenced on induction chemotherapy with intravenous cytarabine (170 mg daily for 7 days) and doxorubicin (50 mg daily for 3 days), alongside supportive care including transfusion support and antimicrobial treatment. Although a partial reduction in gingival swelling was observed, the patient’s clinical condition deteriorated, and he died approximately one week after completion of induction chemotherapy.

## Discussion

Gingival hypertrophy is a recognised manifestation of acute leukaemia and is most frequently associated with monocytic and myelomonocytic subtypes of AML, particularly AML-M5 and AML-M4 [9]. In contrast, gingival involvement in APL, especially the microgranular variant, is uncommon, with only limited case reports described [10, 11]. Reported incidence varies across studies; retrospective data from India suggest rates ranging from 5.7% to 20% [10], while data from African populations remain scarce. Such presentations may be misattributed to non-haematological causes, complicating early recognition.

The microgranular variant of APL is known to exhibit atypical morphological features that can resemble monocytic leukaemias, particularly AML-M4 and AML-M5. Blasts in this variant often demonstrate sparse cytoplasmic granulation and bilobed or folded nuclei, which may overlap with the morphology of monocytic cells. This morphological similarity, combined with clinical features such as gingival hypertrophy more classically associated with monocytic leukaemias, and the absence of overt coagulopathy or hyperleukocytosis in our case, can create diagnostic uncertainty. Furthermore, the immunophenotypic profile (CD13+, CD33+, MPO+) is not specific and may be seen in other myeloid subtypes, including M4 and M5. Therefore, definitive diagnosis requires molecular confirmation of the PML-RARA fusion. The diagnosis of microgranular APL requires integration of morphological features, immunophenotyping, and molecular testing.

The limited published literature on APL presenting with gingival hypertrophy suggests that prognosis can be favourable when the condition is recognised early and treated promptly with ATRA-based therapy. In one reported case, gingival hypertrophy resolved within two weeks of treatment with ATRA and arsenic trioxide, with subsequent complete morphological and molecular remission [10]. In another case, the patient was treated with ATRA and arsenic trioxide and remained clinically well on follow-up [11]. Although the number of reported cases remains very small, these reports suggest that gingival hypertrophy itself does not necessarily confer a poor prognosis; rather, outcome appears to depend largely on early recognition and timely access to appropriate therapy. Our case contrasts with these reports, as resource limitations precluded access to ATRA-based treatment, and the patient had a fatal outcome.

In this case, progressive gingival enlargement and bleeding led to initial dental management before haematological evaluation. While oral complaints appropriately prompt dental assessment, persistence of gingival hypertrophy, particularly when accompanied by bleeding or systemic symptoms, should raise suspicion for an underlying systemic disorder. Early haematological evaluation, including a FBC and peripheral blood film, is therefore essential, and dentists and frontline clinicians play a key role in recognising features that warrant escalation.

APL exhibits clinical and laboratory heterogeneity, particularly in the microgranular variant. Although hyperleukocytosis and coagulopathy are commonly described, our patient did not demonstrate overt DIC or hyperleukocytosis at presentation. Our patient presented with pancytopenia, and the absence of other classical laboratory features, such as hyperleukocytosis or overt coagulopathy, contributed to an atypical clinical presentation and highlights the need to consider APL even when hallmark findings are incomplete.

Early diagnosis of APL is crucial, as prompt initiation of differentiation therapy with ATRA, with or without ATO, has resulted in long-term survival rates of at least 95% in contemporary studies [5]. Current recommendations emphasise initiating ATRA at the first clinical suspicion of APL, even before cytogenetic confirmation, to reduce early mortality [12]. In this case, deviation from guideline-recommended therapy due to resource constraints necessitated treatment with conventional anthracycline- and cytarabine-based induction chemotherapy, which is associated with inferior outcomes in APL. The fatal outcome likely reflects atypical presentation and thus delayed haematological assessment, and limited access to essential targeted therapy rather than disease biology alone.

Overall, this case demonstrates a rare clinical presentation of APL and underscores the importance of clinician awareness, timely haematological assessment of atypical oral presentations, and access to essential therapies in reducing mortality in this otherwise highly curable disease. Gingival hypertrophy may represent an early but misleading manifestation of APL, and atypical clinical features, including the absence of some classical laboratory findings such as hyperleukocytosis or overt coagulopathy, can contribute to diagnostic delay and suboptimal management. Given the aggressive nature of APL, early suspicion, prompt haematological evaluation, and immediate initiation of ATRA at first clinical suspicion remain essential to improving outcomes. Strengthening awareness among dentists and frontline clinicians, alongside improving access to essential diagnostic and therapeutic resources, is important to reducing mortality.
